# Stereotactic body radiotherapy for low-risk prostate cancer: five-year outcomes

**DOI:** 10.1186/1748-717X-6-3

**Published:** 2011-01-10

**Authors:** Debra E Freeman, Christopher R King

**Affiliations:** 1 Naples Radiation Oncology, PA, USA; 2 Department of Radiation Oncology, UCLA School of Medicine, CA, USA

## Abstract

**Purpose:**

Hypofractionated, stereotactic body radiotherapy (SBRT) is an emerging treatment approach for prostate cancer. We present the outcomes for low-risk prostate cancer patients with a median follow-up of 5 years after SBRT.

**Method and Materials:**

Between Dec. 2003 and Dec. 2005, a pooled cohort of 41 consecutive patients from Stanford, CA and Naples, FL received SBRT with CyberKnife for clinically localized, low-risk prostate cancer. Prescribed dose was 35-36.25 Gy in five fractions. No patient received hormone therapy. Kaplan-Meier biochemical progression-free survival (defined using the Phoenix method) and RTOG toxicity outcomes were assessed.

**Results:**

At a median follow-up of 5 years, the biochemical progression-free survival was 93% (95% CI = 84.7% to 100%). Acute side effects resolved within 1-3 months of treatment completion. There were no grade 4 toxicities. No late grade 3 rectal toxicity occurred, and only one late grade 3 genitourinary toxicity occurred following repeated urologic instrumentation.

**Conclusion:**

Five-year results of SBRT for localized prostate cancer demonstrate the efficacy and safety of shorter courses of high dose per fraction radiation delivered with SBRT technique. Ongoing clinical trials are underway to further explore this treatment approach.

## Background

Prostate cancer is thought to have unique radiobiology, characterized by a low α/β ratio relative to surrounding normal tissues [1,2]. A growing body of evidence from clinical studies using hypofractionated radiation provides support that the α/β ratio for prostate cancer is lower than that for the bladder and rectum, and that consequently a therapeutic gain could be achieved using fewer, high-dose fractions (see reviews by Dasu [3] and Macias and Biete [4]). High-dose-rate (HDR) brachytherapy can deliver radiation to a tightly constrained treatment volume using large doses per fraction. Recent multi-institutional findings reported by Martinez et al. for early stage prostate cancer show a 5-year biochemical disease-free survival of about 90% for HDR brachytherapy, which is comparable to their own low-dose-rate (LDR) brachytherapy outcomes, with lower late toxicity levels [5-7].

Stereotactic body radiotherapy (SBRT) has recently emerged as an alternative technique to deliver hypofractionated radiotherapy to the prostate, comparable in many respects to HDR brachytherapy, but with a non-invasive approach [8-14]. The concept is not entirely novel. In the 1980 s, prostate cancer patients were treated in the United Kingdom with 6 fractions of 6 Gy each, delivered over three weeks. Good disease control with no major early or late morbidity was obtained [15]. Innovations in image-guidance technology, the ability to automatically correct for the movement of the prostate during treatment, and delivery of highly-conformal beam profiles have greatly enhanced the capability of delivering high dose fractions to a well-defined target, with sharp dose fall-off towards the bladder and rectum [16-18].

King et al. at Stanford University began treating low-risk prostate cancer patients with the CyberKnife system (Accuray Inc., Sunnyvale, CA) in late 2003, using five fractions of 7.25 Gy (total 36.25 Gy). At a median follow-up of 33 months for the first 41 patients, the urethral/rectal toxicity profile was comparable to that from dose-escalated external beam radiotherapy (EBRT) [12]. Friedland and Freeman et al. in Naples, Florida, began their SBRT program in early 2005, treating low- and intermediate-risk patients with 5 fractions of 7.0 Gy (total 35 Gy). Outcomes from their first 112 patients showed a biochemical control rate of 97% at 24 months median follow-up and toxicity similar to or better than published outcomes of EBRT [9].

Given the intense level of interest in academic and community practices, the ramifications for the management of prostate cancer, and the potential positive economic impact on prostate cancer treatments, we felt it would be both timely and of significant value to examine outcomes from patients with the longest follow-up available to date with the aim of determining disease control and toxicity for SBRT at a median of 5 years. In this report, we present for the first time the results from our combined experience.

## Materials and methods

### Patient Characteristics

The Stanford prostate SBRT program began in December 2003. Eligible patients had newly diagnosed, biopsy-proven prostate cancer presenting with low-risk features. The criteria for low-risk classification included a pre-treatment PSA of 10 ng/mL or less, Gleason score of 3+3 or lower and clinical stage T1c or T2a/b. Patients with a Gleason score of 3+4 were included if present in 2 or fewer cores and involving less than 5 mm aggregate tumor length. Patients with prior treatment (hormone therapy or transurethral resection of prostate) were excluded. The Naples prospective program began in February 2005. Eligibility criteria were similar to that of the Stanford program, except that it included patients with Gleason scores 3+4 in addition to those with Gleason scores of 3+3. For the current study, we included only the Naples patients with Gleason scores of 3+3 or lower, to increase the homogeneity of this combined study population. Staging work-up included a bone scan and CT scan of the abdomen and pelvis. Both centers had IRB-approval for enrolling patients in their clinical trial.

The current patient cohort consists of consecutively treated patients with the longest follow-up participating in the Stanford [12] and Naples studies [9]. Two patients were lost to follow-up within 12 months of treatment and were not included. Two others died of non-prostate cancer related disease at 12 and 51 months after treatment. This study is therefore composed of 41 patients with a median follow-up of 5 years (4.2-6.2 years). The median patient age was 66 years (range 48 to 83 years). The median initial PSA was 5.6 ng/mL (range 0.7 to 10 ng/mL).

### Treatment Planning and Delivery

Three to four gold fiducial markers were placed in the prostate under transrectal ultrasound guidance for image-guided positioning and motion tracking. Treatment planning CT scans were performed at a slice thickness of 1.25 mm, either on the same day (Stanford) or one week after fiducial placement (Naples). MRI scans were obtained for all Naples patients, with preferred sequences of T2* GRE or T1 post Gd, using a slice thickness of 1-2 mm. Planning CTs were used either alone (Stanford) or fused with MRI images (Naples), to differentiate the prostate and the proximal 1 cm of the seminal vesicles (the gross tumor volume, or GTV) from the rectum, urogenital diaphragm, bladder, distal seminal vesicles, and other surrounding structures. The clinical target volume consisted of a 3 mm expansion anteriorly and laterally and a 1 mm posterior expansion. The planning target volume (PTV) consisted of an additional 2 mm expansion anteriorly and laterally and 2 mm posteriorly, to account for errors in target definition and delivery.

All patients were treated with the CyberKnife system, composed of a 6 MV linear accelerator mounted on a robotic arm, with two orthogonal kilovoltage X-ray imagers that provide real-time stereoscopic image guidance and automatic correction for movements of the prostate throughout treatment. Typically, 150-200 non-coplanar beams were delivered in each treatment session. Patient positioning and target tracking were accomplished by registering the location of the fiducials in the real time images to their location in the planning CT. The robot automatically corrected the accelerator's aim to account for both translational and rotational movement of the patient or prostate during the treatment.

Treatment for the Stanford patients consisted of 5 fractions of 7.25 Gy for a total dose of 36.25 Gy. The prescription dose covered at least 95% of the planning target volume, normalized to the 88-92% isodose line. The rectal dose-volume goals were <50% of the rectum receiving 50% of the prescribed dose, <20% receiving 80% of the dose, <10% receiving 90% of the dose, and <5% receiving 100% of the dose. The Naples patients received 5 fractions of 7 Gy each, for a total dose of 35 Gy. The planning objective was also to deliver the prescribed dose to at least 95% of the PTV. For the rectum, the V36 Gy constraint was <1 cm^3^; for the bladder, the V37 Gy was <10 cm^3^. The Stanford rectal dose-volume guidelines were followed whenever possible. Treatments were given over 5 consecutive days for all but 3 patients in the combined cohort.

### Follow-up and Toxicity Scoring

Patients were followed every 3 months during the first year and every 6-12 months thereafter. PSA levels were obtained at each follow-up. Toxicity and quality of life measures for Stanford patients were assessed using the EPIC scale. Naples patients were assessed with the American Urological Association (AUA) and Sexual Health Inventory for Men (SHIM) surveys. Toxicities were subsequently scored based on Radiation Therapy Oncology Group (RTOG) urinary and rectal toxicity criteria [19], and toxicities requiring intervention were noted. (The authors acknowledge that the RTOG scoring system may be insensitive to subtle changes in urinary or bowel function.) Biochemical failure was assessed using the nadir+2 (Phoenix) definition [20].

## Results

### PSA Response

**Figure 1:**
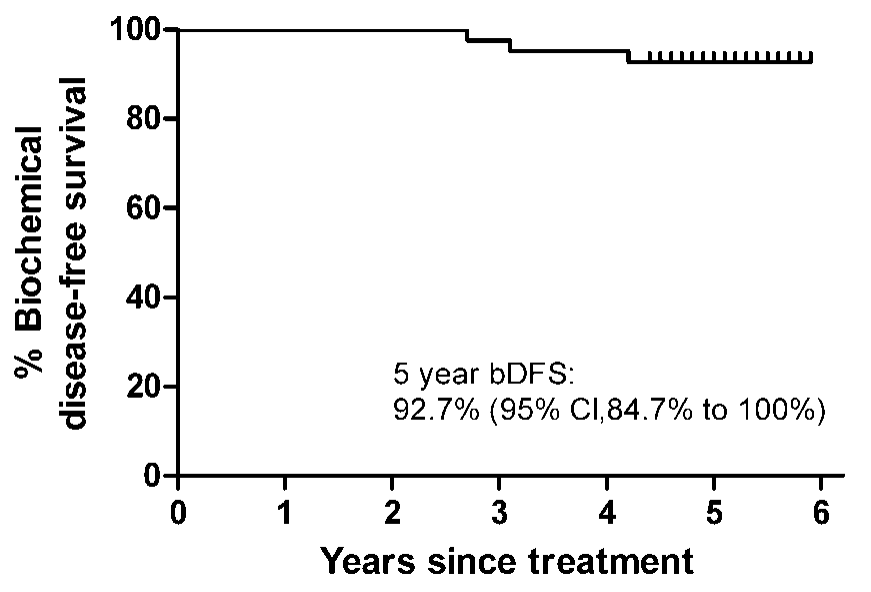
**Kaplan-Meier biochemical disease-free survival curve after SBRT for prostate cancer**. Median follow-up is 5-years. Three of the 41 patients recurred, at 33, 37 and 42 months post-treatment. Tick marks indicate censored patients.

The 5-year biochemical progression-free survival rate was 92.7% (95% CI = 84.7% to 100%, Figure 1). PSA fell from a pre-treatment mean (± SD) of 5.4 ± 2.4 ng/ml to a mean post-treatment value of 0.34 ± 0.35 ng/ml at last follow-up for non-recurring patients. Median PSA nadir was 0.3 ng/ml. Comparing non-recurring Stanford patients (treated with 36.25 Gy) to Naples patients (treated with 35 Gy), the mean PSA at last follow-up was significantly lower for the Stanford group (0.18 ± 0.14 ng/ml vs. 0.51 ± 0.46 ng/ml, *p *= 0.002). The mean follow-up for the Stanford patients was about 4.5 months longer than for the Naples patients (5.17 vs. 4.78 years). Three patients developed biochemical progression at 33, 37 and 42 months, respectively. Two patients received the 35 Gy dose; the third received 36.25 Gy. In each case, biopsy confirmed pathologic evidence of malignancy within the prostate gland and a negative metastatic work-up. The remaining patients continued to have stable or declining PSA levels at last follow-up.

### Toxicity

As previously reported, patients tolerated treatments very well, resuming normal activities within one week of completion. Acute symptoms of dysuria, urinary urgency, frequency, nocturia and/or tenesmus typically resolved within one month of treatment completion. Late toxicities are summarized in Table 1. No patient has experienced grade 3 or greater late rectal toxicity. Only one patient developed late grade 3 urinary toxicity following repeated urologic instrumentation, including cystoscopy and urethral dilatation. No urinary incontinence has been observed. Twenty-five percent of patients reported mild (grade 1) and 7% moderate (grade 2) urinary symptoms following treatment. King et al. [12] previously reported less frequent grade 1-2 urinary toxicity when SBRT treatments were delivered on non-consecutive days (QOD) vs. daily (QD). As the majority of patients in this study received QD treatment, a similar comparison was not possible.

**Table 1:** Late urinary and rectal toxicity on the RTOG scale for prostate cancer patients after SBRT.

RTOG Grade	I	II	III	IV
Urinary	25% (10/41)	7% (3/41)	2.5% (1/41)	0%

Rectal	13% (6/41)	2.5% (1/41)	0%	0%

## Discussion

This report demonstrates that SBRT can achieve high rates of durable disease control for patients with low-risk prostate cancer while resulting in low levels of bladder and rectal toxicity. The current results extend prior independently conducted studies by the authors [9,12], demonstrating the potential of SBRT monotherapy to provide durable disease control with few serious complications in low-risk prostate cancer patients. Our 5-year progression-free survival rate of 93% compares favorably with that obtained with surgery, LDR or HDR brachytherapy [21-26].

In a recent update of the Stanford experience, which included 67 low-risk patients [27], King et al. succinctly reviewed the rationale for hypofractionation in the management of prostate cancer. At a median follow-up of 2.7 years, the PSA relapse-free survival was 94%, and toxicity was equal to or lower than observed in dose-escalation studies. Disease control rates above 90% are entirely consistent with predictions based on an α/β ratio for prostate cancer of 1.5 Gy. Using the linear-quadratic radiobiologic model, 36.25 Gy yields an equivalent dose at 2 Gy per fraction, or EQD2, of 91 Gy for this α/β.

In addition, both disease control and toxicity outcomes with SBRT compare favorably to other treatments for low-risk prostate cancer. In a study comparing outcomes for radical prostatectomy and IMRT to a dose of at least 72 Gy [28], no significant difference in 5-year biochemical disease-free survival (bDFS) rates was detected for low-risk patients (prostatectomy resulted in a bDFS of 92.8% vs. 85.3% for IMRT, *p *= 0.20). Similar 5-year bDFS rates, ranging from 76% to 92% for radical prostatectomy, 69% to 89% for external beam radiotherapy at doses of 66 to 72 Gy, and 83% to 88% for seed brachytherapy, have been reported in retrospective comparisons of these various treatments [21-26]. A recent report of a multi-institutional retrospective study comparing HDR brachytherapy to seed brachytherapy showed bDFS to be about 90% for both modalities. Somewhat higher 5-year bDFS rates, in the 92-95% range, have been obtained in other studies of surgery, high-dose and hypofractionated EBRT, and seed brachytherapy for low-risk patients [29-32]. Thus, the 5-year bDFS of 92.7% obtained in the current study is clearly within the range of disease control expected using modern surgical and high-dose radiation techniques.

In the coming years, the long-term outcomes of several other studies of SBRT for organ-confined prostate cancer will be reported. Katz et al. reported 3-year results on 304 patients with low- and intermediate-risk disease, with favorable outcomes [11]. An update with 42 months median follow up was presented at ASTRO 2010 [33], and 5-year data from this study should be available in 2011. An additional 114 low-intermediate risk prostate patients were treated with SBRT in Naples in 2006, so that data will reach 5-year maturity next year. Acute toxicity from a prospective study underway at the University Hospitals Case Medical Center were presented at the 2009 ASCO meeting [34]. Georgetown has also treated prostate cancer using SBRT; early data were presented at the 2010 ASCO meeting [35]. Two prospective studies funded by Accuray, examining the effects of delivering either a homogeneous, EBRT-like dose distribution or an HDR-like, heterogeneous distribution [10] should complete enrollment in the next 6 months, adding another 600 patients to the collective data pool. A phase III study comparing 12-fraction versus 5-fraction SBRT for localized prostate cancer is currently under review by the RTOG, and a proposed, phase III study from the University of Miami will compare extended fractionation (26 fractions) versus accelerated hypofractionation (5 fractions) for low-intermediate risk disease. As data from these various studies mature, we will develop a clearer picture of long-term outcomes following SBRT.

## Conclusion

The current analysis is the first report of 5-year outcomes of SBRT for low-risk prostate cancer, and biochemical disease control is comparable to other available therapies, with equal to or better toxicity profiles. In addition, the treatment can be completed in a time period that is notably shorter (1-2 weeks) than conventional radiotherapy (8-9 weeks) and neither hospitalization nor surgical recovery is involved. These characteristics of SBRT may benefit patients by reducing travel costs and lost work time, allowing a more immediate return to normal, daily routines, and potentially reducing health care costs. We look forward to future multicenter studies that will examine outcomes with this treatment approach.

## Competing interests

DF has received reimbursement as a consultant/employee for Accuray, Inc.

CK has no financial conflicts of interest.

## Authors' contributions

Both authors contributed equally to the conduct of the study and the contribution of patient data to the analysis. DF conducted analyses and wrote the initial draft of the paper. Both authors read and approved the final manuscript.
